# *In vitro* modelling of alveolar repair at the air-liquid interface using alveolar epithelial cells derived from human induced pluripotent stem cells

**DOI:** 10.1038/s41598-020-62226-1

**Published:** 2020-03-26

**Authors:** Sander van Riet, Dennis K. Ninaber, Harald M. M. Mikkers, Teresa D. Tetley, Carolina R. Jost, Aat A. Mulder, Thijs Pasman, Danielle Baptista, André A. Poot, Roman Truckenmüller, Christine L. Mummery, Christian Freund, Robbert J. Rottier, Pieter S. Hiemstra

**Affiliations:** 1 Department of Pulmonology, Leiden University Medical Center, Leiden, The Netherlands; 2 Department of Cell and Chemical Biology, Leiden University Medical Center, Leiden, The Netherlands; 3 LUMC hiPSC core facility, Leiden University Medical Center, Leiden, The Netherlands; 4 Department of Anatomy and Embryology, Leiden University Medical Center, Leiden, The Netherlands; 5 National Heart & Lung Institute, Imperial College London, London, United Kingdom; 6 Department of Biomaterials Science and Technology, Technical Medical (TechMed) Centre, Faculty of Science and Technology, University of Twente, Enschede, The Netherlands; 7 Department of Instructive Biomaterials Engineering, MERLN Institute for Technology-Inspired Regenerative Medicine, Maastricht University, Maastricht, The Netherlands; 8 Department of Pediatric Surgery, Erasmus MC-Sophia Children’s Hospital, Rotterdam, The Netherlands

**Keywords:** Induced pluripotent stem cells, Chronic obstructive pulmonary disease, Experimental models of disease

## Abstract

Research on acute and chronic lung diseases would greatly benefit from reproducible availability of alveolar epithelial cells (AEC). Primary alveolar epithelial cells can be derived from human lung tissue but the quality of these cells is highly donor dependent. Here, we demonstrated that culture of EpCAM^+^ cells derived from human induced pluripotent stem cells (hiPSC) at the physiological air-liquid interface (ALI) resulted in type 2 AEC-like cells (iAEC2) with alveolar characteristics. iAEC2 cells expressed native AEC2 markers (surfactant proteins and LPCAT-1) and contained lamellar bodies. ALI-iAEC2 were used to study alveolar repair over a period of 2 weeks following mechanical wounding of the cultures and the responses were compared with those obtained using primary AEC2 (pAEC2) isolated from resected lung tissue. Addition of the Wnt/β-catenin activator CHIR99021 reduced wound closure in the iAEC2 cultures but not pAEC2 cultures. This was accompanied by decreased surfactant protein expression and accumulation of podoplanin-positive cells at the wound edge. These results demonstrated the feasibility of studying alveolar repair using hiPSC-AEC2 cultured at the ALI and indicated that this model can be used in the future to study modulation of alveolar repair by (pharmaceutical) compounds.

## Introduction

Alveolar injury resulting in decreased lung function is a hallmark of various acute and chronic lung diseases. These include acute respiratory distress syndrome (ARDS), emphysema in chronic obstructive pulmonary disease (COPD) and idiopathic pulmonary fibrosis^1,2^. The alveolar epithelium consists of flattened type 1 alveolar epithelial cells (AEC1) that allow gas exchange and the cuboidal type 2 alveolar epithelial cells (AEC2) that produce surfactant, mediate host defence and act as local progenitors for the AEC1^2,3^. These AEC are a primary target for inhaled substances; injury to these cells is considered a major initiating event for many lung diseases, including those above. Endogenous repair has been shown to contribute to recovery following injury, but in chronic lung diseases these repair processes are either insufficient or impaired^4–8^. This results in cumulative damage to the alveolar compartment and subsequently reduced diffusion capacity and lung function. Treatments aimed at alveolar repair and improving the alveolar barrier integrity are urgently needed but none are yet available. Targeting signalling pathways that have been shown to be involved in lung repair, including bone morphogenetic protein (BMP)/transforming growth factor-β (TGF-β), Hedgehog, fibroblast growth factor (FGF), Notch and Wnt signalling^6,9^, might be attractive therapeutic options. Notably among these, Wnt/β-catenin signalling is impaired in AEC in COPD as well as in experimental emphysema in mice^7,8,10^.

For the development of therapies that target AEC, a human (preferable patient-specific) *in vitro* alveolar repair model would be of great benefit. Tumour cell lines (A549), immortalized AEC1 and primary AEC are currently most widely used for *in vitro* studies^11,12^. However, immortal cell lines do not fully capture the complexity of the alveolar epithelium. Primary human AEC2 (pAEC2) can be isolated from resected lung tissue but nearly all patients undergoing lung surgery have an underlying disease that affects the yield and function of the isolated cells, making them less than ideal for large-scale screening or direct extrapolation of outcomes to other conditions^13^. The availability of normal lung tissue, e.g. from non-diseased human lungs otherwise discarded as unsuitable for lung transplantation, is limited. Furthermore, fetal lungs, which could also be a source of AEC, may not be ideal to study repair of adult lung tissue. Importantly, the use of pAEC2 is further complicated by their inability to undergo passage in culture and tendency to differentiate spontaneously to terminally differentiated AEC1 confounding their use in lung repair studies^14^.

Since their initial description in 2007, human induced pluripotent stem cells (hiPSC) have been intensely used to study development and disease *in vitro*, and more recently for toxicity screening and drug discovery^15^. hiPSC lines can be generated from any healthy individual or patient and thus potentially represent an unlimited source of tissue-specific cells, which can be used as *in vitro* models for screening effectiveness or toxicity of candidate therapeutic agents. Human AEC cultures have been successfully derived from human embryonic stem cells^16,17^ and from hiPSC previously^18–26^. These latter studies relied on directed differentiation of hiPSC into the endodermal lineage using Activin A, followed by differentiation of this definitive endoderm into foregut endoderm through inhibition of TGF-β and BMP signalling. An essential next step was the development of NKX2-1^+^ lung progenitors using a mixture of growth factors, that can be directed to an alveolar fate by continued culture on tissue culture plastic or embedding in an extracellular matrix as organoids^18,22,24^. Although, hiPSC-derived lung epithelial cells have been used for disease modelling^27^, they have not yet been used to study alveolar repair. The aim of the present study was to investigate the feasibility of using hiPSC-derived AEC2 (iAEC2) cultured at the air-liquid interface (ALI) as an *in vitro* model to study alveolar repair and to compare this model with that using pAEC2 isolated from lung tissue.

## Materials and Methods

### hiPSC maintenance and differentiation into alveolar epithelial cells

The hiPSC lines LUMC0044iCTRL44.9 and LUMC0065iCTRL08 were generated and characterized at the LUMC hiPSC core facility from female skin fibroblasts^28^ or from erythroblasts derived from a healthy male donor using lentiviral^29^ or episomal vectors^30^, respectively. The cells were maintained under fully defined serum-free conditions on vitronectin- (StemCell Technologies, Vancouver, Canada) coated 6-well tissue culture dishes (Corning, Corning, NY) in mTeSR1 medium (StemCell Technologies). The cells were passaged weekly (1:15 split ratio) using “Gentle Cell Dissociation Reagent” (StemCell Technologies).

**Figure 1:**
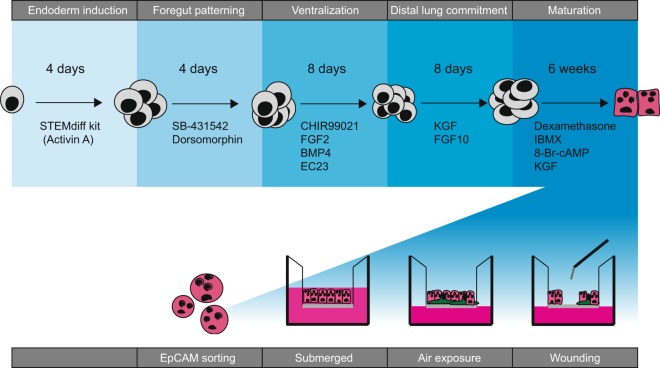
Overview of human induced pluripotent stem cell (hiPSC) differentiation into alveolar-like cells and culture at the air-liquid interface. The various steps followed to achieve differentiation of hiPSC towards an alveolar fate is schematized. Following 4 weeks of maturation, the cells are sorted based on EpCAM expression and seeded on the Transwell insert for further maturation and culture at the air-liquid interface. See supplement for details.

iAEC2s were generated from hiPSCs by stepwise recapitulation of fetal lung development as shown schematically in Fig. 1, and outlined in the Results. A detailed description of the culture method and key reagents is listed in the online Supplement.

### Isolation and culture of primary alveolar epithelial cells

pAEC2 were isolated from tumour-free lung tissue of patients undergoing lung resection at the Leiden University Medical Center (LUMC, The Netherlands). The use of surplus lung tissue for research following surgery was within the framework of patient care and in line with the “Human Tissue and Medical Research: Code of conduct for responsible use” (2011) (www.federa.org) and followed advice of the LUMC Medical Ethical Committee. Tissue donation was based on a no-objection system for coded anonymous use of waste tissue, left-over from diagnostic or therapeutic procedures. “No-objection” negates the need for individual informed consent and was approved by the IRB. All methods were carried out in accordance with relevant guidelines and regulations. pAEC2 were isolated and cultured essentially as described^13^. Briefly, resected lung tissue was cut into pieces of approximately 5 cm^3^ and incubated with 10 ml trypsin (0.25% w/v in Hanks’ Balanced Salt Solution) for 15 min; this was repeated multiple times for a total incubation time of 45 min. Trypsin was then blocked using newborn calf serum (NBCS) (Sigma-Aldrich, St. Louis, MO). The tissue was cut into smaller pieces in the presence of NBCS and DNase (Sigma-Aldrich), and passed through strainers of different sizes to obtain single cells in suspension. These were plated in uncoated T75 flasks for 2 hours at 37 °C with serum-free DCCM-1 (Biological Industries, Kibbutz Beit-Haemek, Israel) medium to allow attachment of most non-AEC2. Following 2 hours of attachment, non-attached (pAEC2) cells were collected and seeded on 1% Purecol- (Advanced Biomatrix, Carlsbad, CA) coated Transwell inserts with 0.4 µm diameter pores (Corning). The pAEC2 were maintained in DCCM-1 with 10% (v/v) NBCS.

### Quantitative real-time PCR

RNA isolation was performed using Maxwell tissue RNA extraction kit (Promega, Leiden, The Netherlands) according to the manufacturer’s protocol. RNA concentrations were determined using the NanoDrop ND-1000 Spectrophotometer (NanoDrop Technologies, Wilmington, DE) and cDNA was synthesized by reversed transcription of 1 μg of RNA, using oligo-dT primers (Qiagen), dNTP (Promega), and M-MLV Polymerase (Promega) in the presence of RNasin (Promega). Quantitative real-time PCR (qPCR) was conducted using IQ SYBR green supermix (Bio-Rad, Hercules, CA) and a CFX-384 real-time PCR detection system (Bio-Rad). qPCR reactions were performed using the primers shown in Table 1. Reference genes OAZ1 and ATP5B were selected using the NormFinder method^31^. Bio-Rad CFX manager 3.1 software (Bio-Rad) was used to calculate the arbitrary gene expression using the Livak method.

**Table 1:** Primer sequences.

Gene	Forward (5′ to 3′)	Reverse (3′ to 5′)
*SOX2*	TGGACAGTTACGCGCACAT	CGAGTAGGACATGCTGTAGGT
*FOXA2*	ACTACCCCGGCTACGGTTC	AGGCCCGTTTTGTTCGTGA
*CPM*	GCGCTGGATTTCAACTACCAC	TCCCGCCCAACAGTCTCAT
*NKX2-1*	AGCACACGACTCCGTTCTC	GCCCACTTTCTTGTAGCTTTCC
*SOX17*	GTGGACCGCACGGAATTTG	GGAGATTCACACCGGAGTCA
*SOX9*	AGCGAACGCACATCAAGAC	CTGTAGGCGATCTGTTGGGG
*SFTPB*	CTTCCAGAACCAGACTGACTCA	GCTCGGAGAGATCCTGTGTG
*SFTPC*	CTCTCTGCAGGCCAAGCCCG	TTCCACTGACCCGGAGGCGT
*SFTPD*	CCTTACAGGGACAAGTACAGCA	CTGTGCCTCCGTAAATGGTTT
*OAZ1*	GGATCCTCAATAGCCACTGC	TACAGCAGTGGAGGGAGACC
*ATP5B*	TCACCCAGGCTGGTTCAGA	AGTGGCCAGGGTAGGCTGAT
*PDPN*	AACCAGCGAAGACCGCTATAA	CGAATGCCTGTTACACTGTTGA

### Immunofluorescence

Cells were fixed in 4% paraformaldehyde for 1 hour at 4 °C, 100% methanol was added for permeabilization for 10 min at 4 °C, and non-specific binding sites were blocked in blocking solution consisting of 5% (w/v) BSA and 0.3% (v/v) Triton X100 in PBS. Between each step, cells were washed 3 times with PBS. Subsequently the cells were incubated overnight at 4 °C with primary antibody (Table 2) diluted in blocking solution. Next, filters were washed 3 times in PBS and stained with secondary antibody, and DAPI as nuclear staining. Secondary antibodies (Table 2) and DAPI were also diluted in blocking solution and incubated in the dark for 1 hour at room temperature. Afterwards, filters were washed 3 times with PBS and mounted on microscope slides using ProLong gold antifade (Thermo Fisher). Images were made using a Leica TCS SP8 confocal inverted microscope (Leica Microsystems, Wetzlar, Germany) and processed using the Leica Application Suite Advanced Fluorescence software (LAS AF, Leica Microsystems).

**Table 2:** Antibodies used for immunofluorescence.

Protein	Company	Cat#	Dilution
*Primary antibodies*			
NKX2-1	Novus biological	NBP2-29434	1:100
Pro-SFTPC	R&D	H00006440-D01P	1:200
LPCAT1	Atlas antibodies	HPA012501	1:100
Podoplanin	Abcam	ab10288	1:100
*Secondary antibodies*			
Alexa 568 donkey-anti-mouse	Invitrogen	A10037	1:200
Alexa 488 goat-anti-rabbit	Invitrogen	A11008	1:200
Alexa 568 donkey-anti-rabbit	Invitrogen	A10047	1:200

### Transepithelial electrical resistance

The epithelial barrier function of epithelial monolayer cultures was determined by measuring the transepithelial electrical resistance (TEER) using the MilliCell-ERS (Millipore, Bedford, MA). TEER was expressed as Ω*cm^2^.

### Electron microscopy

Cells cultured on filter were fixed by adding double concentrated fixative 1 on 1 to the culture medium (3% GA/0.2 M cacodylate, final concentration 1.5% GA/0.1 M cacodylate buffer). After an 1 hour fixation cells were rinsed with 0.1 M cacodylate buffer and postfixed with 1% OsO4/1.5% ferricyanide/0.1 M cacodylate buffer. After rinsing with cacodylate buffer a second postfixation with 0.5% RuO4/MQ was performed. After rinsing with cacodylate buffer the filter with the fixed cells was carefully cut out from the Transwell, cut in small pieces of about 1 × 2 mm and dehydrated up to ethanol 70%, followed by mixtures of EPON LX-112 and ethanol 70%. The filter pieces were positioned in a mold, allowing cross sections of the filter. After polymerization of the EPON, 80 nm ultrathin sections were made, and after staining with uranyl acetate and lead citrate, the sections were examined with an electron microscope (FEI Tecnai T12 Twin, 120 kV). Overlapping images were collected and stitched together into separate images as previously described^32^.

### Wound healing assay

A circular wound assay was performed as described in^33^. Briefly, 500 μl PBS was added to the apical surface of the ALI-cultured AEC2 to facilitate mechanical wounding. The wound was applied by using a sterile Pasteur pipette with a soft tip to scrape the cell layer, creating a wound with a diameter of 3 mm. After wounding, the apical surface of the cultures was washed with 200 μl PBS to remove cellular debris. Wound healing was assessed by light microscopy, and images were taken at different time points. Images were analysed using NIH-ImageJ software (v1.50i).

### Data analysis

Statistical analysis was performed in GraphPad PRISM 6.0 (GraphPad Software Inc., La Jolla, CA). Data was analysed using one-way ANOVA with Tukey correction, or for wound closure with two-way ANOVA multiple comparison test. Error bars depict SEM. Differences were considered significant at p < 0.05.

## Results

### Generation of induced type 2 alveolar epithelial cells (iAEC2s) from hiPSC

#### Selection of reference genes for monitoring differentiation

**Figure 2:**
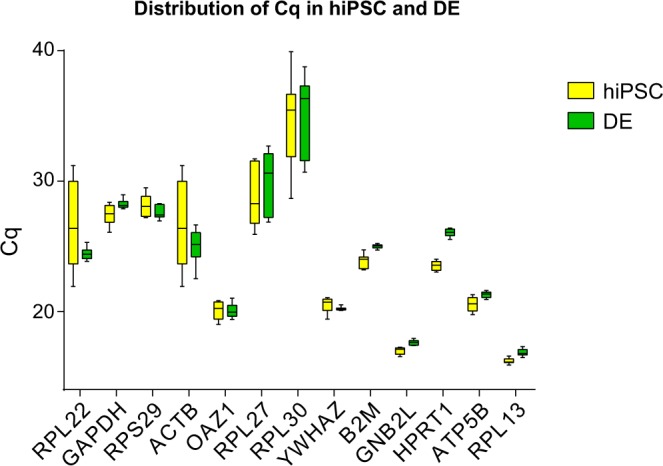
Identification of suitable reference genes using NormFinder. Expression value of commonly used reference genes showing the threshold values obtained by qPCR. Expression in LUMC0044iCTRL44.9 and LUMC0065iCTRL08 and derived definitive endoderm. (n = 8 independent experiments).

Gene expression is commonly used to monitor hiPSC differentiation since the cells undergo extensive phenotypic changes which can be monitored accurately using cell-type specific reference genes. To identify the most suitable reference genes, we performed NormFinder analysis at the first step of differentiation^31^. The variation of expression stability of 13 candidate reference genes in hiPSC and definitive endoderm (DE) cells was analysed. Figure 2 shows the distribution of Cq values of 13 reference genes (Supplemental Table 1) in our hiPSC lines and their corresponding differentiated DE derivatives. Table 3 shows the stability values of all candidate reference genes as well as the inter-group variation. The three genes with the highest stability were *ATP5B* (0.243), *GNB2L* (0.278) and *OAZ1* (0.281), of which the former and latter were chosen for further studies. Notably, other frequently used reference genes like *GAPDH, B2M and ACTB* showed either poor stability values or high Cq, making them less suitable for monitoring differentiation.

**Table 3:** Evaluation of reference gene stability using NormFinder in hiPSC and DE.

Gene name	Stability value	Intergroup variation	hiPSC	DE
*ATP5B*	0.243	ATP5B	0.061	0.161
*GNB2L*	0.278	GNB2L	0.205	0.252
*OAZ1*	0.281	OAZ1	0.118	0.029
*RPL13*	0.305	RPL13	0.246	0.323
*GAPDH*	0.355	GAPDH	0.795	0.061
*B2M*	0.372	B2M	0.089	0.117
*YWHAZ*	0.393	YWHAZ	0.063	0.158
*RPS29*	0.556	RPS29	0.966	0.268
*RPL27*	0.625	RPL27	1.248	1.934
*RPL30*	0.786	RPL30	4.020	3.831
*HPRT1*	0.804	HPRT1	0.120	0.033
*ACTB*	0.845	ACTB	3.498	0.402

#### Development of differentiation protocol

**Figure 3:**
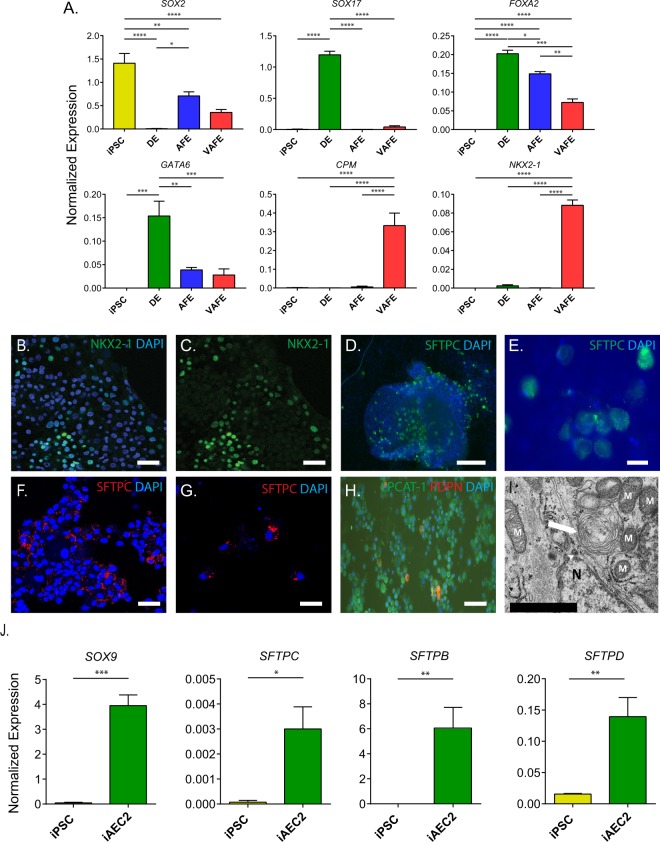
Differentiation of human induced pluripotent stem cells towards an alveolar fate. (**A**) Expression of differentiation-associated genes in undifferentiated hiPSC, DE, AFE and VAFE (n = 6 separate experiments from 2 cell lines). (**B–I**) Representative images of cells at various differentiation stages stained for (**B**,**C**) NKX2-1 scale bar = 50 µm, (**D**,**E**) Pro-surfactant protein C expression pre-EpCAM sorting, scale bar = 50 µm and 20 µm respectively (**F**,**G**) Pro-surfactant protein C expression post-EpCAM sorting, scale bar = 50 µm (**H**) LPCAT-1 (green) Podoplanin (Red), scale bar = 50 µm Nuclei (blue) stained with DAPI. (**I**) Electron microscopic image of lamellar body in iAEC2 cells following 2 weeks of ALI culture (n = 1) N = Nucleus, M = mitochondria, Arrow = lamellar body, scale bar = 1 µm. (**J**) Normalized gene expression of genes associated with a distal lung and alveolar fate (n = 3 independent experiments). Data shown as mean; error bars represent SEM, *p < 0.05, **p < 0.01, ***p < 0.001, ****p < 0.0001.

An overview of the final differentiation protocol based on optimized procedures is shown in Fig. 1 and described in detail in the online supplement. Differentiation was monitored using markers described as being associated with different stages of differentiation as shown in Fig. 3 and SFig. 1. hiPSC were first plated in 6-well plates and directed towards DE. 4 days later, DE cells were present as evidenced by decreased expression of pluripotency genes and increased expression of the endodermal markers SOX17 and FOXA2 (Fig. 3A). DE cells were exposed to the SMAD2/3 inhibitor SB-431542 and the BMP inhibitor dorsomorphin to induce anterior foregut endoderm (AFE). Dorsomorphin resulted in a stronger increase in expression of AFE markers compared to treatment with the recombinant BMP inhibitor Noggin (SFig. 1). Successful generation of AFE was confirmed after 4 days of differentiation based on the decreased expression of the endodermal marker SOX17, maintenance of FOXA2 expression, and increased SOX2 expression (Fig. 3A)^34^. Following AFE induction, formation of ventral anterior foregut endoderm (VAFE) was induced using FGF2, BMP4, CHIR99021 (canonical Wnt-activator) and EC23 (retinoic acid pathway agonist). Following 8 days of differentiation directed towards VAFE, increased expression of NKX2-1 and CPM was observed (Fig. 3A), which have been described as the earliest markers of lung development expressed in the VAFE^22,35^. Expression of NKX2-1 at this stage of differentiation was markedly enhanced by the presence of FGF2, and this was confirmed at the protein level by immunofluorescent staining of NKX2-1 (Fig. 3B,C). Expression was observed in a subset of cells, indicating a heterogeneous population. Subsequently, the cells were directed to a lung progenitor phenotype expressing SOX9 and ID2 by 8 days of exposure to KGF and FGF10 (SFig. 3). Following progenitor differentiation, the cells were exposed to dexamethasone, KGF, IBMX and 8-Br-cAMP for 6 weeks to direct cells to an AEC2 phenotype. The development of AEC2-like cells was verified by immunofluorescent staining (Fig. 3D,E) and mRNA expression of surfactant proteins (Fig. 3J).

In order to obtain a more enriched cell population, after 4 weeks of alveolarization EpCAM^+^ cells were isolated by MACS-based sorting, and the cells were next seeded on a Transwell insert and cultured for 2 weeks at the air-liquid interface (ALI). Expression of pro-surfactant protein C (pro-SFTPC) was assessed using fluorescent staining (Fig. 3F,G). Following selection and 2-week ALI culture of EpCAM^+^ cells, the majority of the cells stained weakly positive for the lamellar body-associated enzyme Lysophosphatidylcholine acyltransferase 1 (LPCAT-1) (Fig. 3H). Remarkably, after 2 weeks of culture the expression of surfactant protein C was maintained and expression of podoplanin (PDPN) was detected in some cells. PDPN was not detected directly following EpCAM isolation, suggesting a loss of type 2 phenotype and an increased expression of protein classically associated with type 1 phenotype during ALI culture. Staining of EpCAM-isolated cells showed 64.7% (n = 3, SEM = 2.53) positive for NKX2-1 (SFig. 2). To explore contamination with other lineages, we analysed expression of genes associated with other endodermal and non-endodermal lineages during the differentiation process (SFig. 3). We found that none of the markers analysed were expressed at higher levels than in pAEC2. Furthermore, staining for various airway epithelial cell-associated cellular markers (TP63, FOXJ1, SCGB1A1) indicated these were not present in our cultures (data not shown). We further validated the final differentiation protocol for generation of iAEC2 by using electron microscopy to confirm the presence of lamellar bodies (Fig. 3I).

### Comparison of primary and induced AEC2 cultured at the air-liquid interface

**Figure 4:**
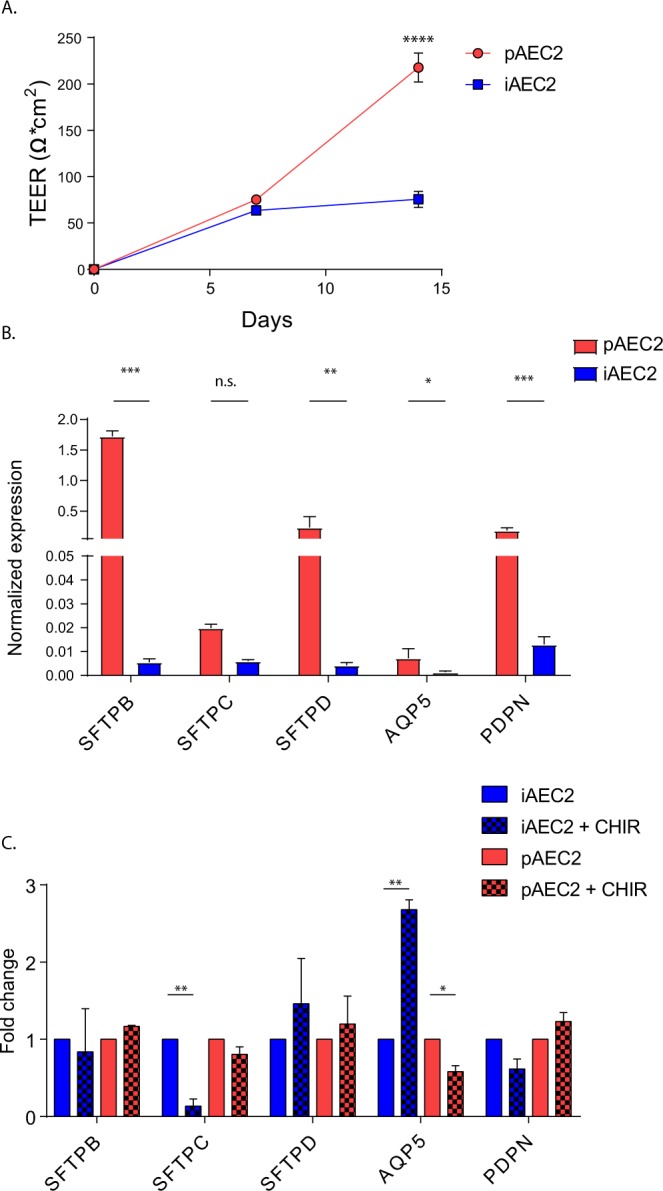
Comparison of hiPSC-derived and isolated AEC2 cells. (**A**) Change of TEER over 2 weeks of culture at the air-liquid interface (n = 3 independent experiments from 2 cell lines). (**B**) Normalized expression of markers associated with AEC2 and AEC1 phenotype following 2 week culture at the air-liquid interface (n = 7). (**C**) Normalized expression alveolar associated markers following 2 weeks of culture at the air-liquid interface and following stimulation with CHIR99021 (n = 3). SFTPB/C/D = Surfactant protein B/C/D, AQP5 = Aquaporin 5, PDPN = Podoplanin. Data shown as mean; error bars represent SEM, *p < 0.05, **p < 0.01, ***p < 0.001, ****p < 0.0001.

We next compared characteristics of cultured iAEC2 and pAEC2. To this end, EpCAM-sorted iAEC2 and pAEC2 were seeded on Transwell inserts to allow culture at the ALI. The cells grew to confluence in approximately 2 weeks. They then formed a functional barrier that prevented leakage of medium from the basal compartment upon removal of the medium from the apical compartment. Establishment of this barrier was confirmed by assessing TEER. This showed that pAEC2 increased their barrier over the 2-week culture period to 200 Ω*cm^2^ (Fig. 4A). In contrast, TEER values of iAEC2 reached a plateau of 80 Ω*cm^2^ after 1 week of culture. AEC2 were cultured for 2 weeks at the ALI and expression of the AEC2-associated markers surfactant protein B, C and D (SFTPB, SFTPC and SFTPD), aquaporin 5 (AQP5) and PDPN, markers associated with an AEC1 phenotype, were analysed at the mRNA level (Fig. 4B). Whereas iAEC2 cells showed overall lower levels of expression of AEC2 markers compared to pAEC2 cells, their expression was detected at the protein level using fluorescent staining. In order to determine if the epithelial cell maintenance could be improved by canonical Wnt activation, we stimulated the ALI cultured cells for 1 week with the Wnt-activator CHIR99021. Culture of iAEC2 cells with CHIR99021 caused a significant decrease in expression of SFTPC and an increase of AQP5, suggesting bias towards an AEC1 phenotype (Fig. 4). In contrast, in the pAEC2, no significant effect of CHIR99021 on expression of AEC markers was noted.

### Circular wound closure assay

**Figure 5:**
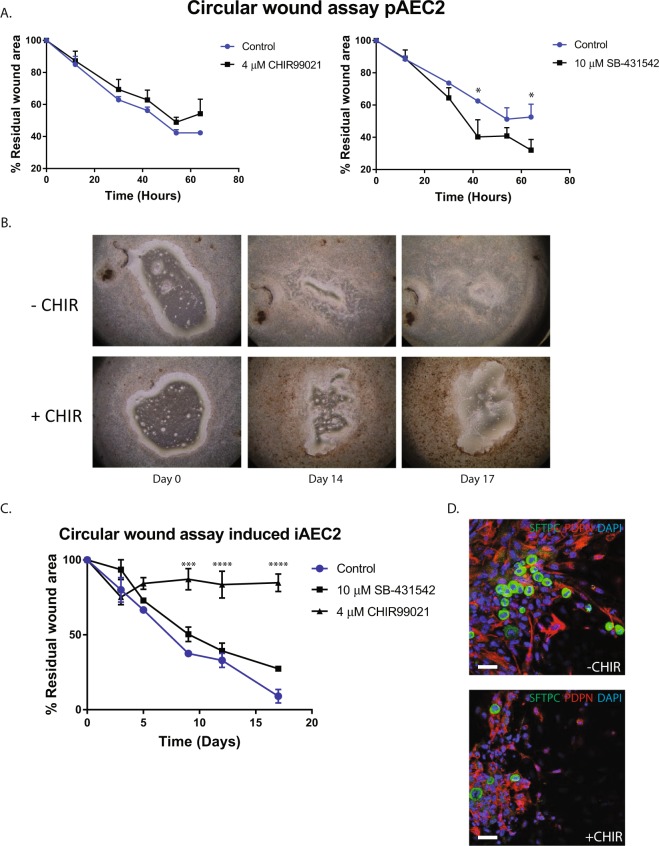
Wound closure assay at the air-liquid interface. (**A**) Quantification of wound closure of isolated pAEC2 cells stimulated with SB-431542 or CHIR99021 (n = 3). (**B**) Representative images of wound closure of iAEC2 at the air liquid interface. (**C**) Quantification of wound closure of iAEC2 stimulated with SB-431542 or CHIR99021 (n = 3). (**D**) Wound edge of iAEC2 during wound closure. SFTPC (Green), Podoplanin (Red). Data shown as mean; error bars represent SEM, *p < 0.05, ***p < 0.001, ****p < 0.0001.

To study repair in the alveolar epithelial ALI model, a circular mechanical wound was created by scraping pAEC2 layers cultured at the ALI. In this assay, epithelial wound closure was monitored over time showing that under control conditions full wound closure was achieved between 72 and 84 hours. Exposure to the Wnt-activator CHIR99021 following wounding caused a non-significant decrease in wound closure rate, whereas exposure to the TGF-β signalling inhibitor SB-431542 significantly increased the rate of wound closure (Fig. 5A). Microscopic analysis suggested that in the early phases of wound repair, cells migrated into the wound area and displayed an elongated morphology. These observations suggested that wound closure is achieved by a mix of proliferation and migration. Notably, in cultures exposed to CHIR99021, more elongated cells were observed at the wound edge, which appeared to delay wound closure (Fig. 5D).

Similar wound closure assays were then performed on iAEC2. Closure was slower compared to that observed with pAEC2 and full wound closure was only achieved at day 17. This is in line with our observation that the growth rate of iAEC2 was markedly lower than of pAEC2. Also in line with the observations with pAEC2, exposure of iAEC2 to CHIR99021 following injury reduced wound closure compared to control, and for iAEC2 this effect was statistically significant. Interestingly, in contrast to pAEC2, SB-431542 did not affect iAEC2 wound closure compared to control.

In line with our findings in pAEC2, CHIR99021 also caused accumulation of elongated cells at the wound edge in iAEC2 cultures (Fig. 5B). These cells were further characterized by staining for pro-SFTPC and PDPN. The results showed that CHIR99021 treatment increased the number of PDPN^+^ cells while decreasing the number of pro-SFTPC^+^ cells at the wound edge, indicating a change in the number of cells retaining AEC2-like phenotype (Fig. 5D). Whether this phenotypical change contributes to the decrease in wound closure, requires further investigation.

## Discussion

In this study, we demonstrated that selection of EpCAM^+^ cells from differentiating hiPSC and followed by ALI culture generates hiPSC-derived AEC2-like cells (iAEC2) suitable for studying alveolar wound repair. The introduction of an EpCAM-based sorting step during the maturation phase resulted in a more homogeneous population of pro-SFTPC^+^ cells that contained lamellar bodies evident by electron microscopy. When analysing wound closure at the ALI, we found that the Wnt-activator CHIR99021 delayed wound closure in both pAEC2 and iAEC2 cultures, with this effect being more pronounced and significant in iAEC2. In iAEC2 cultures, and not in pAEC2 cultures, this treatment was accompanied by an increase in cells positive for PDPN, a marker associated with an AEC1 phenotype, with a concomitant decrease in pro-SFTPC positive cells at the wound edge.

Before our study, other groups had reported the generation of AEC2 from embryonic-^16,17^ and induced pluripotent stem cells^18–26^. Our protocol was largely based on these published protocols, with some modifications that improved purity and maturity in our hiPSC cell lines. Notably, our differentiation protocol required FGF2 for a robust induction of NKX2-1 expression during the ventralization step. Another difference with published protocols is that we isolated EpCAM^+^ cells after 4 weeks of maturation of distal progenitor cells in the dexamethasone mixture. EpCAM^+^-sorted cells were further matured for 2 weeks before assessing AEC2-associated marker expression. However, based on NKX2-1 staining, the isolated EpCAM^+^ population appeared heterogeneous which may be partly because EpCAM is not an alveolar-specific marker. Other studies included sorting at earlier stages of differentiation on the basis of carboxypeptidase M (CPM) surface expression or using NKX2-1 or pro-SFPTC reporter constructs, to enhance the efficiency and homogeneity of AEC2 population^22,23^. We reasoned that, compared to these approaches, EpCAM has the advantage of being an easily accessible cell surface protein expressed at the last stage of differentiation. Furthermore, using surface EpCAM as a marker does not require introduction of reporter constructs in hiPSC lines, facilitating wider application when using multiple hiPSC lines. Until recently, many studies have relied on the expression of pro-SFTPC at the mRNA or protein level to determine AEC2 identity but immature AEC2 may also express pro-SFTPC, whereas lamellar bodies as examined here by electron microscopy are a characteristic of mature AEC2^36^. In addition, we obtained indirect evidence for the presence of these lamellar bodies by staining for the lamellar body-associated protein LPCAT-1. An important and acknowledged drawback in the use of hiPSC-derived cells is their heterogeneity with respect to maturation, even when using optimized protocols^37,38^. In our cultures, we have also assessed markers of airway epithelial cells and non-lung epithelial cells in view of the fact that it is known that NKX2-1 cells are plastic and may differentiate to other lung and non-lung phenotypes^39^. Expression of these markers was absent or not higher compared to primary alveolar cells. Although our iAEC2 cells displayed markers and functions typical of pAEC2, it was unclear whether additional cues are required for these cells to fully mature and become identical to AEC2 *in situ* in lung tissue. Single cell RNAseq analysis, epigenetic and other –omics based analyses of the iAEC2 might provide more information on the maturation state of subgroups of cells in the population. This was clearly demonstrated in a recent report by Hurley *et al*.^39^, showing how the use of single cell transcriptomics, computational modelling and lineage tracing using DNA barcoding can be applied to further improve differentiation of hiPSC-derived lung progenitors towards alveolar epithelial cells.

In an effort to mimic the structural orientation of the alveoli more closely, we cultured the cells on Transwell inserts to allow air exposure. Our culture setup demonstrated the feasibility of culturing and maintaining both the pAEC2 and iAEC2 at the ALI. This setup is not only more physiological than using submerged cultures as it mimics the luminal air exposure in the alveolus, but importantly it also allows exposure to airborne substances such as inhaled toxicants and micro-organisms through a more anatomically accurate route. Furthermore, the effect of change in conditions as following birth when submerged cells are suddenly air-exposed or in ARDS when air-exposed cells are suddenly submerged in fluid as a result of vascular leakage, could be studied in this setup.

Interestingly, it was reported that in hiPSC-derived AEC2 cultures only few cells that express proteins associated with an AEC1 phenotype are detected^35^, which is in marked contrast to cultures of pAEC2 from adult lung tissue that rapidly lose surfactant and other AEC2-associated markers *in vitro*^14^. This may suggest that the pathway driving spontaneous transdifferentiation in adult pAEC2 is not (yet) as active in iAEC2. Interestingly, we observed that treatment of iAEC2 cultures at the ALI with the Wnt-activator CHIR99021 decreased the number of cells with AEC2 characteristics while increasing AEC1-associated markers. Whether our findings are related to the use of ALI culture, is unclear at present. Furthermore, we cannot firmly conclude that we have obtained AEC1-like cells based on the loss of AEC2 markers and the upregulation of PDPN or AQP5. In our alveolar wounding model, we noted that this appearance of iAEC1-like cells was most pronounced at the wound edge. These findings on the effect of CHIR99021 are in apparent contrast to a recent mouse study, which suggested that Wnt signalling is required to maintain stemness of AEC2 cells, and that Wnt signalling prevents transdifferentiation of AEC2^40,41^. In addition, Xu *et al*. previously showed that the canonical Wnt ligand Wnt3a blocked transdifferentiation of AEC2 to AEC1^42^. To add to the complexity of Wnt signalling during generation of hiPSC-derived AEC2, it was demonstrated that temporary withdrawal of CHIR99021 is associated with iAEC2 maturation^23,39^, whereas maintenance of proliferation requires adding back CHIR99021^39^. A note of caution is needed, since CHIR99021 activates Wnt signalling through GSK3beta inhibition, and its mode of action is therefore not specific. Tamo *et al*.^26^ recently reported that the Wnt-signalling inhibitor IWT increased transdifferentiation of iAEC2 to iAEC1 under submerged conditions, further pointing to a possible role of experimental design and agents used to study AEC transdifferentiation. Another mechanism that has been implicated in transdifferentiation of AEC2 to AEC1 is the autocrine action of TGF-β^43^, which may be antagonized by BMP signalling^44^. Using the TGF-β inhibitor SB-431542, we noted that the wound closure in iAEC2 cultures was unchanged compared to control, suggesting that TGF-β induced transdifferentiation does not play a dominant role in wound closure of iACE2 at the ALI.

The marker profile of iAEC2 was largely similar to that of pAEC2 but there were differences. First, expression levels of AEC2-specific markers were lower in iAEC2. Second, in our wound model, closure of iAEC2 wounds was notably slower than that of pAEC2, which might partly be explained by differences in culture media. Whereas pAEC2 are maintained in a rich, new-born calf serum supplemented medium, iAEC2 are grown in a fully defined medium which is suitable for maintenance, but possibly suboptimal for expansion. Another important difference between iAEC2 and pAEC2 is that the latter were derived *in situ* in the presence of supporting cells, whereas similar cells were absent during differentiation of iACE2 from hiPSC. Our pAEC2 isolation protocol yielded a highly enriched population, but we cannot exclude minor contamination with mesenchymal cells. Various studies have shown the ability of mesenchymal cells^22,25^ as well as endothelial cells^45^ to support alveolar epithelial cell development. Miller recently reported on the generation of a bud tip progenitor-like population of cells by culturing hiPSC-derived NKX2-1^+^ ventral foregut spheroids in presence of FGF7, CHIR99021 and retinoic acid^46^. These cells maintained their multi-lineage potential and upon engraftment into murine airways developed into cells with characteristics of alveolar and airway cells. We did not use supporting cells during the generation of alveolar epithelial cells from hiPSC. Furthermore, in our current model of wound repair, we studied closure of alveolar epithelial wounds in the absence of other cell types that might influence closure rates. *In vivo* AEC are in close proximity to the surrounding endothelial cells allowing efficient gas transfer. Therefore, a limitation of our model is that endothelial cells, as well as other alveolar structural cells and for example intraluminal macrophages are absent. Furthermore, whereas AEC2 in lung tissue are exposed to the mechanical forces of breathing, these forces are absent in our model. It has been described that stretching of alveolar cells can contribute to maturation and surfactant secretion (44). *In vivo* mechanical cues play a crucial role in organ formation and development. The differentiation pressure that could be provided by stretch in a lung-on-chip model could provide a more realistic alveolar environment and possibly more homogenous populations.

A major advantage of using hiPSC lines for the development of disease-relevant models, is that it allows the possibility of generating various differentiated cell types from the same cellular and genetic source, for example, cells with endothelial functions expressing endothelial markers (CD31, CD144, VWF)^47,48^. We are currently exploring which media combinations are suitable for long-term co-culture of AEC2 and endothelial cells, either derived from resected lung tissue or hiPSC. The cell types that can be generated from hiPSC are not limited to AEC2 or endothelial cells, and with the continuous development of differentiation protocols, the generation of other cells present in the lung parenchyma becomes reality.

In summary, our study provides support for the use of hiPSC in generating relatively mature populations of AEC2 and both confirms and extends previous studies in this area^18,20,22,23,26,35,39^. In addition, the study shows that iAEC2 can be used to study epithelial repair processes. This is relevant for future studies to evaluate drugs or cells that may enhance alveolar repair, an unmet medical need in regenerative medicine.

## Supplementary information

Supplementary Information

Supplementary Figures

## Data Availability

The datasets of the current study are available from the corresponding author upon reasonable request.
