# Supplementary material for: In vitro modelling of alveolar repair at the air-liquid interface using alveolar epithelial cells derived from human induced pluripotent stem cells
Source: Sci Rep. 2020 Mar 26;10:5499. doi: 10.1038/s41598-020-62226-1 (PMC7099095; doi:10.1038/s41598-020-62226-1)
Supplement: Supplementary file 1 — Supplementary Information [file 41598_2020_62226_MOESM1_ESM.docx]

***In vitro* modelling of alveolar repair at the air-liquid interface using alveolar epithelial cells derived from human induced pluripotent stem cells**

Sander van Riet^1^, Dennis K. Ninaber^1^, Harald M. M. Mikkers^2,3^, Teresa D. Tetley^5^, Carolina R. Jost^2^, Aat A. Mulder^2^, Thijs Pasman^6^, Danielle Baptista^7^, André A. Poot^6^, Roman Truckenmüller^7^, Christine L. Mummery^4^, Christian Freund^3,4^, Robbert J. Rottier^8^, Pieter S. Hiemstra^1^

^1^Department of Pulmonology, ^2^Department of Cell and Chemical Biology, ^3^LUMC hiPSC core facility, ^4^Department of Anatomy and Embryology, Leiden University Medical Center, Leiden, The Netherlands; ^5^National Heart & Lung Institute, Imperial College London, London, United Kingdom; ^6^Department of Biomaterials Science and Technology, Technical Medical (TechMed) Centre, Faculty of Science and Technology, University of Twente, Enschede, The Netherlands; ^7^Department of Instructive Biomaterials Engineering, MERLN Institute for Technology-Inspired Regenerative Medicine, Maastricht University, Maastricht, The Netherlands; ^8^Department of Pediatric Surgery, Erasmus MC-Sophia Children's Hospital, Rotterdam, The Netherlands

Address correspondence to:

Sander van Riet/Pieter Hiemstra

Department of Pulmonology, B2-P

Leiden University Medical Center

P.O. Box 9600

2300 RC Leiden, The Netherlands

e-mail: [s.van_riet@lumc.nl](mailto:s.van_riet@lumc.nl); p.s.hiemstra@lumc.nl

**SUPPLEMENTARY MATERIALS AND METHODS**

**Table S1. Key reagents used for generation of iAEC2**

| **Compound** | **Concentration** | **Company** | **Cat#** |
| --- | --- | --- | --- |
| Y-27632 | 10 µM | Cayman Chemical | 10005583-10 |
| SB-431542 | 10 µM | Sigma | S4317 |
| Dorsomorphin | 2 µM | Sigma | P5499-5MG |
| CHIR99021 | 2 µM | Sigma | SML1046-5MG |
| FGF2 | 100 ng/ml | Miltenyi Biotech | 130-104-925 |
| BMP4 | 30 ng/ml | Life technologies | PHC9534 |
| EC23 | 50 nM | Bio-Techne | 4011 |
| FGF7 | 50 ng/ml | Tebu-Bio | 100-19 B |
| FGF10 | 100 ng/ml | Miltenyi Biotech | 130-093-850 |
| Dexamethasone | 50 nM | Sigma | D4902-25MG |
| IBMX | 100 µM | Sigma | I5879 |
| 8-Br-cAMP | 100 µM | Sigma | B5386-5MG |

**Table S2. Primer sequences candidate reference genes**

| Gene | Forward (5’ to 3’) | Reverse (3’ to 5’) |
| --- | --- | --- |
| RPL22 | TCGCTCACCTCCCTTTCTAA | TCACGGTGATCTTGCTCTTG |
| GAPDH | TTCCAGGAGCGAGATCCCT | CACCCATGACGAACATGGG |
| RPS29 | GCACTGCTGAGAGCAAGATG | ATAGGCAGTGCCAAGGAAGA |
| ACTB | TGCGTGACATTAAGGAGAAG | TGAAGGTAGTTTCGTGGATG |
| OAZ1 | GGATCCTCAATAGCCACTGC | TACAGCAGTGGAGGGAGACC |
| RPL27 | ATCGCCAAGAGATCAAAGATAA | TCTGAAGACATCCTTATTGACG |
| RPL30 | ACAGCATGCGGAAAATACTAC | AAAGGAAAATTTTGCAGGTTT |
| YWHAZ | ACTTTTGGTACATTGTGGCTTCAA | CCGCCAGGACAAACCAGTAT |
| B2M | GACCACTTACGTTCATTGACTCC | CAGGGTTTCATCATACAGCCAT |
| GNB2L | GAGTGTGGCCTTCTCCTCTG | GCTTGCAGTTAGCCAGGTTC |
| HPRT1 | AACCCTGTTGTCAATGCCTC | AACACTTCGTGGGGTCCTTTTC |
| ATP5B | TCACCCAGGCTGGTTCAGA | AGTGGCCAGGGTAGGCTGAT |
| RPL13A | AAGGTGGTGGTCGTACGCTGTG | CGGGAAGGGTTGGTGTTCATCC |

**Generation of induced (i)AEC2 from hiPSC**

Directed differentiation of hiPSC into AEC2-like cells was achieved by guiding the cells through sequential embryonic developmental stages. hiPSC colonies were grown and maintained on vitronectin (StemCell Technologies, Vancouver, Canada) coated (10 µg/ml) 6 well plates (Corning, Corning, NY) and cultured in mTESR1 media (StemCell Technologies). The cells were passaged weekly 1:15 using Gentle Cell Dissociation reagent (StemCell Technologies). Differentiation into definitive endoderm was achieved using the STEMdiff definitive endoderm kit (StemCell Technologies) according to the manufacturer’s instructions. Single cell suspensions were obtained by using Gentle Cell Dissociation reagent at room temperature. After cell counting, 2x10^6^ cells were seeded in vitronectin-coated 6 well plates. During the first 24 hours, 10 µM Y-27632 (Cayman Chemicals, Ann Arbor, MI) was added. The following day the medium was changed to endoderm medium, supplemented with compound A and B (StemCell Technologies), and for the next 3 days with addition of only supplements B. Following endoderm induction, cells were dissociated using TrypLE select (ThermoFisher, Waltham, MA) at room temperature and split in a 1:4 ratio for culture in 6-well plates pre-coated with vitronectin. The cells were maintained in serum-free basal differentiation medium, consisting of IMDM (ThermoFisher), Ham’s F12 (ThermoFisher) containing B27 (Invitrogen, Carlsbad, CA), N2 (Invitrogen), 0.1% (w/v) bovine serum albumin Fraction V (Invitrogen), Glutamax (ThermoFisher), and primocin (Invivogen, San Diego, USA). To next achieve anterior foregut endoderm (AFE) induction, the medium was supplemented with 10 µM SB-431542 (Sigma-Aldrich, St. Louis, MO) and 2 µM dorsomorphin (Sigma). During the first day of AFE formation, medium was supplemented with 10 µM Y-27632. Using our cell lines, we found that use of dorsomorphin resulted in a more robust induction of SOX2 compared to use of the recombinant protein noggin (SFig 1). After 4 days of anteriorization, the cells were washed and medium switched to ventralization medium for 8 days for induction of ventral anterior foregut endoderm (VAFE). Ventralization medium is basal medium supplemented with 2 µM CHIR99021 (Sigma-Aldrich), 30 ng/ml recombinant human BMP4 (Life Technologies), 100 ng/ml recombinant human FGF2 (Miltenyi Biotech, Bergisch Gladbach, Germany) and 50 nM EC23 (Bio-Techne, Minneapolis, MN). During the first day, 10 µM Y-27632 was added. Importantly, we found that FGF2 was required in our protocol to achieve a robust expression of NKX2-1. This was observed both when using AFE obtained using noggin (Supplementary Figure 1, middle) as well as that obtained using dorsomorphin (Supplementary Figure 1, lower part). Following 8 days of ventralization the cells were washed and dissociated using TrypLE select at room temperature and split in a 1:2 to 1:4 ratio. The cells were maintained in basal differentiation medium supplemented with 50 ng/ml recombinant human FGF7 (Tebu-Bio, Heerhugowaard, The Netherlands) and 100 ng/ml recombinant human FGF10 (Miltenyi Biotech) for 8 days with 10 µM Y-27632 present during the first 24 hours. After 8 days, the cells were washed and medium replaced with alveolarization medium, consisting of basal differentiation medium supplemented with 50 ng/ml recombinant human FGF7, 50 nM dexamethasone (Sigma-Aldrich), 100 µM 3-Isobutyl-1-methylxanthine (IBMX; Sigma-Aldrich) and 100 µM 8-Bromoadenosine 3',5'-cyclic monophosphate (Sigma-Aldrich). After 4 weeks of “alveolarization” the cells were sorting using MACS beads coated with anti-EpCAM (Miltenyi Biotech) according to the manufacturer’s instructions. The isolated cells are cultured on semipermeable Transwell membranes with 0.4-μm pore size (Corning Costar, Cambridge, MA). Transwells were coated with a mixture of 30 μg/ml PureCol (Advanced BioMatrix, San Diego, CA), 10 μg/ml BSA (invitrogen) and 10 μg/ml fibronectin (Alfa Aeser, Karlsruhe, Germany) in PBS, at 37°C, 5% CO_2_. Once confluent, the apical medium was removed and the cells were exposed to air. On the insert and at ALI the cells where maintained in the “alveolarization” medium.

**Legends to supplementary Figures.**

**Supplemental figure 1. Overview of early optimization.** Results show expression of various markers at the different stages of differentiation, including a comparison of the effect of three compounds mixes (SB [SB 431542] combined with Nog [Noggin]; the Wnt inhibitor IWR-1, or Dors [dorsomorphin]) on AFE generation(second line of figures). The lower four lines of figures show a comparison of VAFE obtained using AFE generated using the best two of these three mixes. RA, retinoic acid analogue EC23; +FGF, +FGF2, CHIR, RA and BMP4. See text for details.

**Supplemental figure 2. Staining of NKX2-1 and EpCAM.** Images depicting staining of NKX2-1 and EpCAM on cytospins of EpCAM-isolated iAEC2 cells.

**Supplemental figure 3.** Gene expression levels of other endodermal or non-endodermal lineages at the various stages of the differentiation process compared to primary AEC2 cells. iAEC2: cells cultured for 2 weeks at ALI following EpCAM isolation. The following markers were assessed: CDX1, HNF4a, NKX6-1, PAX8, PDX1, Thyroglobulin (TG), PAX6, NKX2-1, ID2 and SOX9.
