# Supplementary material for: In vitro modelling of alveolar repair at the air-liquid interface using alveolar epithelial cells derived from human induced pluripotent stem cells
Source: Sci Rep. 2020 Mar 26;10:5499. doi: 10.1038/s41598-020-62226-1 (PMC7099095; doi:10.1038/s41598-020-62226-1)

In vitro modelling of alveolar repair at the air-liquid interface using alveolar epithelial cells derived from human induced pluripotent stem cells

Sander van Riet[1], Dennis K. Ninaber[1], Harald M. M. Mikkers[2,3], Teresa D. Tetley[5], Carolina R. Jost[2], Aat A. Mulder[2], Thijs Pasman[6], Danielle Baptista[7], André A. Poot[6], Roman Truckenmüller[7], Christine L. Mummery[4], Christian Freund[3,4], Robbert J. Rottier[8], Pieter S. Hiemstra[1]

[1]Department of Pulmonology, [2]Department of Cell and Chemical Biology, [3]LUMC hiPSC core facility, [4]Department of Anatomy and Embryology, Leiden University Medical Center, Leiden, The Netherlands; [5]National Heart & Lung Institute, Imperial College London, London, United Kingdom; [6]Department of Biomaterials Science and Technology, Technical Medical (TechMed) Centre, Faculty of Science and Technology, University of Twente, Enschede, The Netherlands; [7]Department of Instructive Biomaterials Engineering, MERLN Institute for Technology-Inspired Regenerative Medicine, Maastricht University, Maastricht, The Netherlands; [8]Department of Pediatric Surgery, Erasmus MC-Sophia Children's Hospital, Rotterdam, The Netherlands

Address correspondence to:

Sander van Riet/Pieter Hiemstra

Department of Pulmonology, B2-P

Leiden University Medical Center

P.O. Box 9600

2300 RC Leiden, The Netherlands

e-mail: s.van_riet@lumc.nl; p.s.hiemstra@lumc.nl

Supplementary figure 1

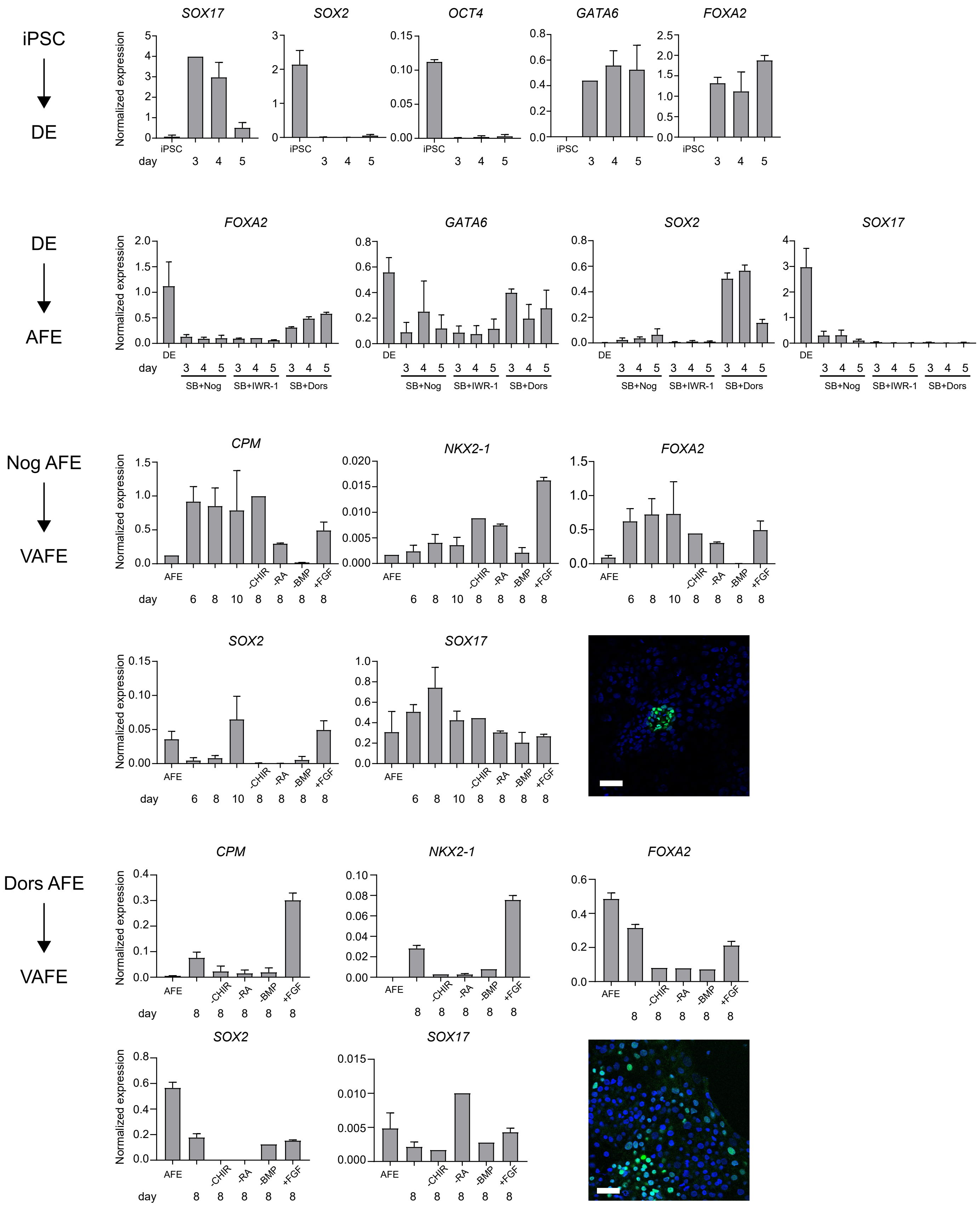

Supplementary figure 2

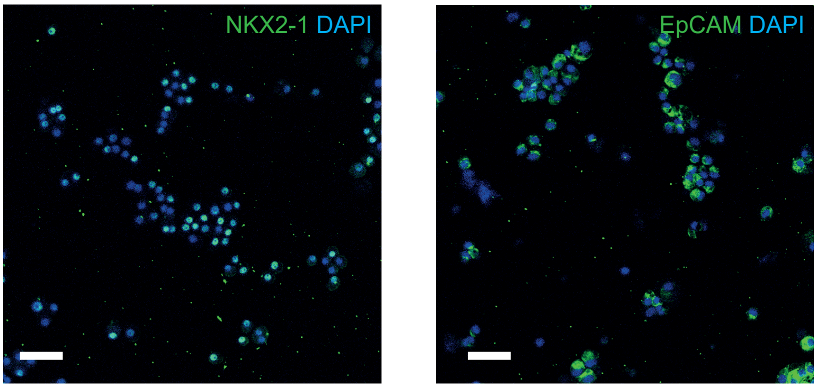

Supplementary figure 3

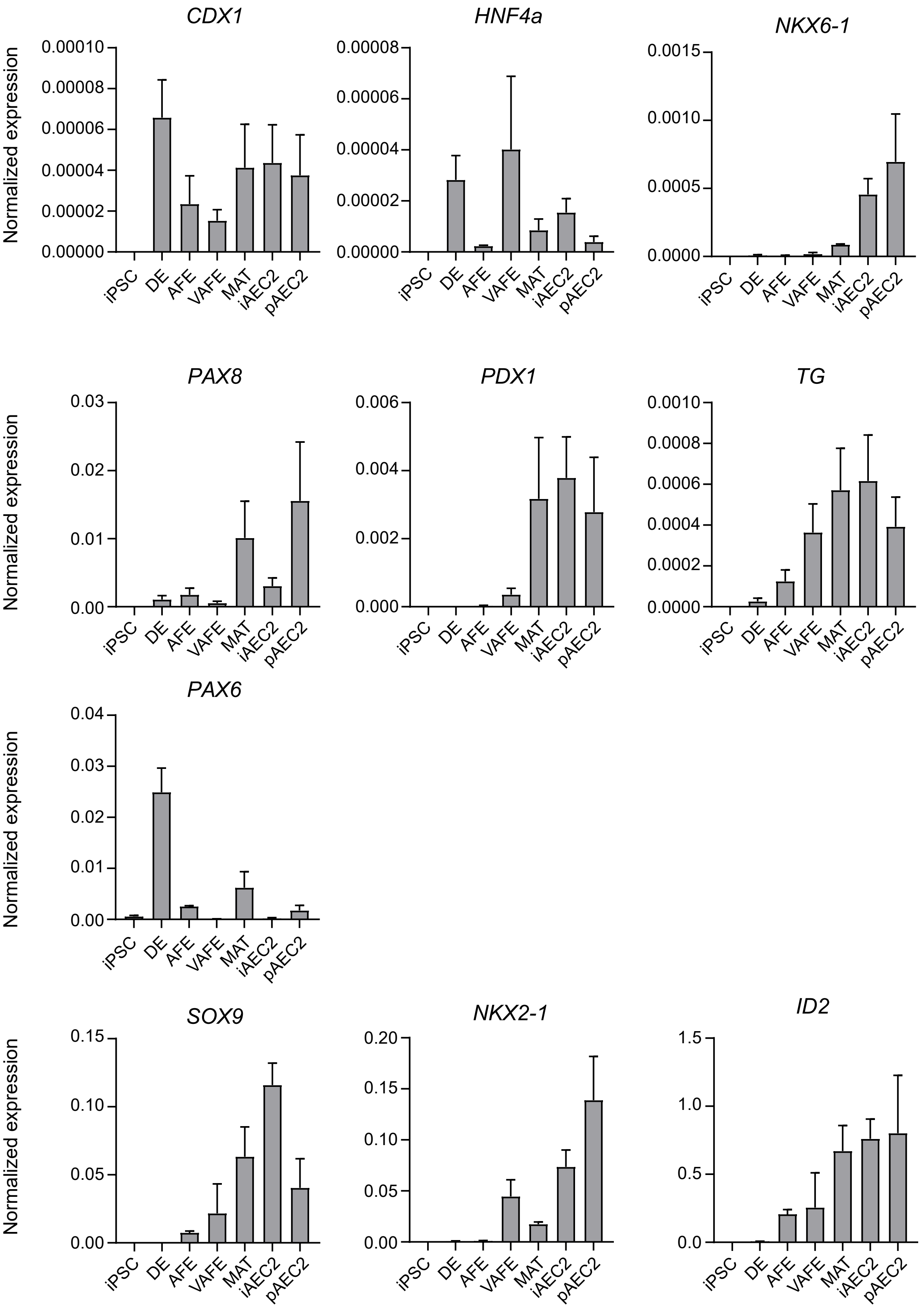

Supplement: Supplementary file 2 — Supplementary Figures [file 41598_2020_62226_MOESM2_ESM.pdf]
